# Sustainability transition for Indian agriculture

**DOI:** 10.1038/s41598-023-34092-0

**Published:** 2023-05-05

**Authors:** Bino Paul, Kamal Kumar Murari, Unmesh Patnaik, Chandra Sekhar Bahinipati, Subash Sasidharan

**Affiliations:** 1 Tata Institute of Social Sciences, Mumbai, India; 2 Indian Institute of Technology Tirupati, Chindepalle, India; 3 Indian Institute of Technology Madras, Chennai, India

**Keywords:** Environmental sciences, Environmental social sciences

## Abstract

Farming in India faces a sustainability challenge due to its overreliance on chemical inputs. For every US$ 1,000 investment in sustainable farming, a US$ 100,000 subsidy is allocated for chemical fertilizers. Indian farming system is far off the optimal nitrogen efficiency, calling for substantial reforms in policy towards the transition to sustainable inputs. We examine the propensity of Indian farmers to adopt biofertilizers and other sustainable inputs. While small farmers are inclined towards chemical inputs, sustainable inputs are costly. Here we show that less than 5 per cent of the farming population contributes to the 95 per cent usage of the bio-fertilizer in India. However, small and marginal farmers contribute substantially to food security. Shifting from chemical to sustainable inputs calls for autonomous investment by the state to augment the capacity and improve affordability. We illustrate the transition to sustainability through a framework that includes scale, affordability, and sustainable inputs.

## Introduction

India’s progress in crossing the threshold towards a sustainable linkage between farming and food security is slow. It failed to move from the Sustainable Nitrogen Management Index (SNMI) precarious zone to a safer zone^1,2^. Although the share of agriculture in the Gross Domestic Product (GDP) has been declining over the decades, it still generates close to two-fifth of employment while its share in the national income is nearly one-fifth^3,4^. Most Indian farms (85%) are marginal and small, relying on the monsoon^5^. Fertilizer is a crucial input in farming^6^. Broadly, fertilizers are of two types: chemical and bio. However, the relationship between fertilizer and soil health is not unidirectional but non-linear^7–9^. Although the scientific literature posits improvements in yield due to chemical fertilizers, there is no dearth of inferences pointing to emerging disadvantages^7,10,11^. The question of optimal fertilizer use is rather difficult for an individual farmer to answer unless they have access to knowledge inputs^12–14^. Although the advocacy for the chemical regime emerges from an angle of food security, it is now increasingly regarded as a threat to sustainability. Sustainability in agriculture refers to increasing yield per unit without negatively affecting soil and water, and non-agricultural sectors^15^. Society encounters two types of challenges: (1) adopting sustainable agricultural practices to feed people now and in the foreseeable future and (2) doubling food production to meet the required demand across the world by 2050^16^. A pertinent question is the pace of transition. The path is not linear. Instead, it requires resources and public goods. A discrete transition from chemical to bio inputs may debilitate the region’s ability to coordinate the public distribution system. Hence, the challenge is to have a balanced transition which is inclusive. Therefore, in this paper, we examine the propensity of Indian farmers to adopt bio-fertilizers and other sustainable inputs.

Although bio inputs are associated with efficiency and return, there are capacity constraints in production, distribution, storage and quality^17,18^. Fundamentally, the problem involves two dimensions: capacity and usage. In India, capacity is yet to come up for a balanced transition that absorbs marginal to medium land holdings^17,19–21^. Compared to the large subsidy for chemical inputs, the public investment in bio inputs is much lower than the threshold. The chemical fertilizer subsidy in India is worth Rs. 1,400 billion (US$ 18 billion), whereas the total allocation for organic inputs is only Rs. 13.2 billion (US$ 0.17 billion)^3,22^. In other words, for every US$ 1,000 investment in sustainable farming, a US$ 100,000 subsidy is allocated for chemical fertilizers. However, recent policy initiatives like the Paramparagat Krishi Vikas Yojana (PKVY) aim to promote farming units to use bio inputs and provide financial support and provisioning of inputs. Such policy interventions may be helpful in terms of cost reduction, even though they may induce a slight revenue decline. Studies have shown that compared to conventional techniques, farms which adopt organic inputs yield better efficiency^23,24^. However, significant challenges emanate from inordinate delays in provisioning financial resources, inadequate training, and lack of scientific facilities^25^.

Although the capacity side generated considerable scholarly interest, studies on the usage of bio inputs in India's context are nascent^25,26^. Therefore, exploring the relationship between scale heterogeneity and the propensity to use bio inputs is crucial. For this purpose, we structure farming units into bins, bagging small, medium and large units in distinctive groups, to gauge the variation in chemical inputs and the adoption of bio inputs using nationally representative farmer-level microdata^27^. Our empirical analysis examines four farming systems focusing on sustainability and scale. Further, we discuss the transition in input usage across the systems. An example is transitioning from low-scale and low-sustainable input to low-scale and high-sustainable input usage.

## Results

### The burden of inputs on farming households

**Figure 1:**
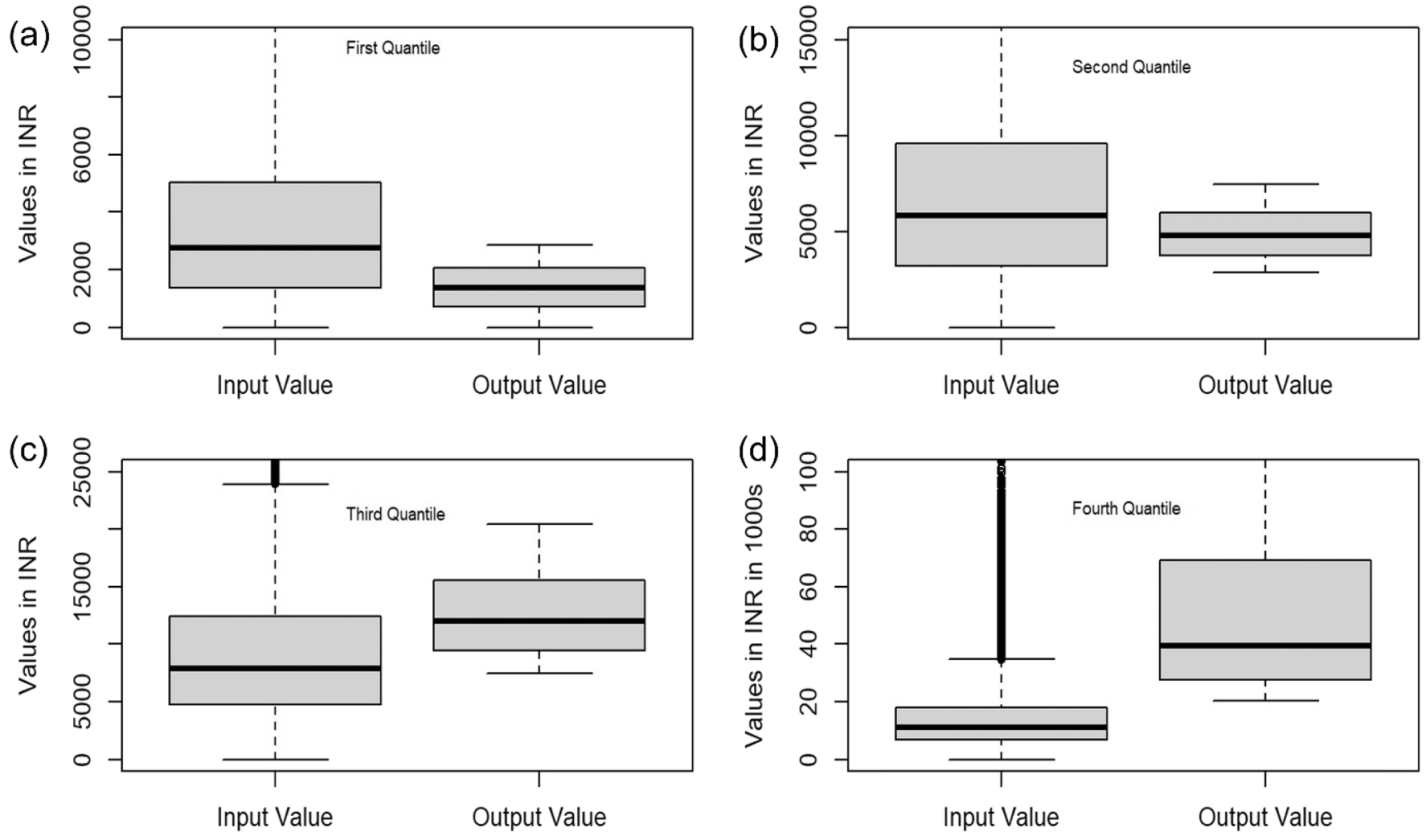
Box and whiskers plot for input and output value (defined in terms of Gross Value Outputs-GVO) values for per unit land cultivated across 58,035 farming households in NSS 77th round survey. The vertical axis in panels (**a**), (**b**), (**c**), and (**d**) are values (in INR) of input and GVO per unit hectare of land. The quartiles in panels (**a**), (**b**), (**c**) ,and (**d**) are based on GVO per unit land.

The performance of the farms in the Indian agriculture sector is sensitive to the scale of value and quality of inputs and gross value of outputs (GVO). GVO is a measure of sales or revenue from products and by-products. To examine GVO across production classes, we split the farming households into four quartiles based on GVO per unit of land cultivated. Box-whisker plots in Fig. 1 visualize each quartile for input and GVO.

Figure 1 shows the input and output distribution per land across four quartiles of farming households based on GVO per ha of land. For the first quartile (Fig. 1a), the distribution of unit input is free of outliers, although the upper bound is high. Most of the area in the box is above the median, implying a marked variability within it. However, the unit output distribution is compact. The median value of GVO is substantially lower than that of the unit input, which implies that the visible variation in the input box coexists with a fixed lower output margin. It is typical of Indian agriculture, which describes the precarious incomes of small farm households in India^28^. The second quartile is not discernibly different from the previous one (Fig. 1b), but the median values of both inputs and output are higher than the first quartile (Fig. 1a). The third quartile turns out to be a changed scenario (Fig. 1c). It features a positive margin between unit output and input per unit of land. While some data points in the input box are outliers, most of the box is above the median. There are no outliers for the unit output, and the above-median area is higher than the lower part. The fourth quartile is entirely different from the rest. The GVO is visibly higher than the input, suggesting positive returns for larger farmers (Fig. 1d). It also confirms the extent of the agrarian crisis in India, as widely acknowledged by various studies^29^. Therefore, in order to overcome the agrarian crisis, the key is lower input costs, sustainable farming methods, and livelihood security for farmers. Further, we observe a similar trend for inputs and GVO in absolute terms across farming classes (Fig. S1).

### A comparative analysis of farm sustainability across household classes

**Figure 2:**
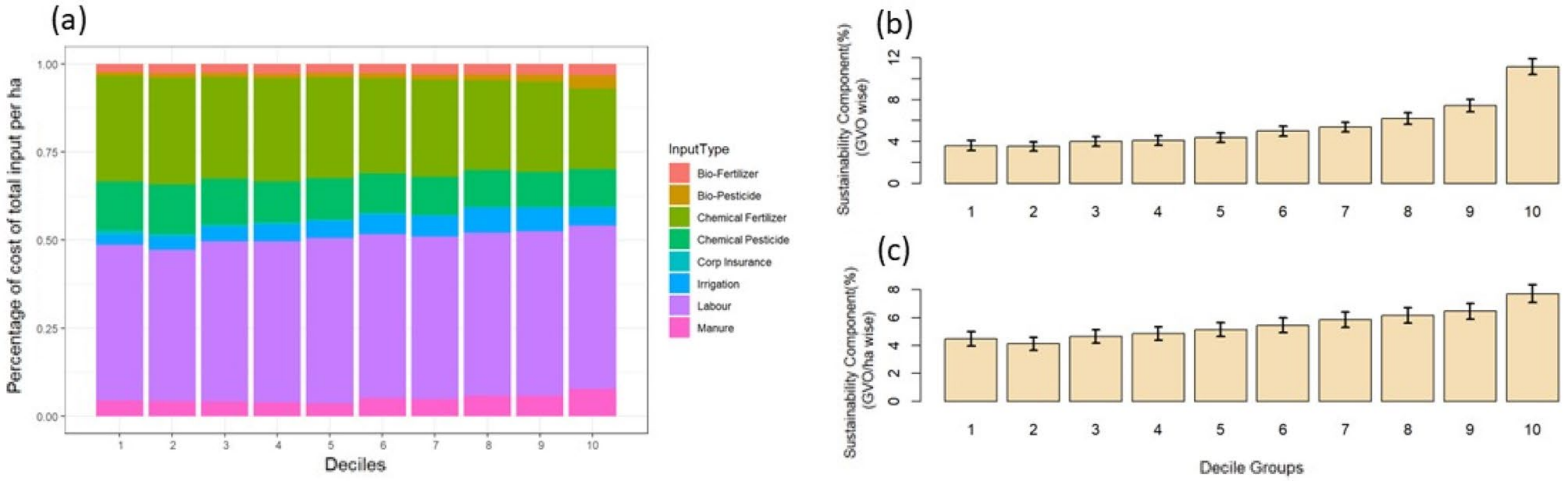
Sustainability component for the input used in Indian farming. The sustainability component is defined as the cost of the ratio of the organic form of input (bio-fertilizer, bio-pesticide, and manure) to the total organic and chemical inputs (fertilizer and pesticides). Panel (**a**) shows the relative cost of different components of input costs per unit area. Note that the cost in panel (**a**) shows only the cost of inputs, labour, crop insurance, and irrigation. The Figure does not consider other inputs such as seeds, machines, land rent, and miscellaneous costs. The value indicates the median values of the farming households in the NSS 77th round. Panels (**b**) and (**c**) shows the sustainability component arranged in deciles of households according to the GVO (panel **b**) and GVO per unit of land (panel **c**). The bar in panels (**b**) and (**c**) shows the average values, and the error bar in the panels shows the minimum and maximum range of values.

An important dimension is the composition of inputs (both in quality and quantity) used in agriculture. The expenditure on agricultural inputs in India consists of improved seeds, fertilizers, crop protection (chemical and biological), machinery, irrigation, land rent, payments for extension, crop insurance premiums, and other miscellaneous expenditures. We reinvestigate the input cost in terms of chemical fertilizer, pesticide, biofertilizer, manure, biopesticide, labour, irrigation and crop insurance by inscribing it in the deciles of the GVO (Fig. 2a). Interestingly, we observe a divergence between chemical inputs and green inputs. In the case of chemical fertilizers and pesticides, we observe a consistent decline in their share as the decile increases. However, the pattern reverses for green components that consist of biofertilizer, manure and biopesticide. It implies that large farmers are more inclined towards green inputs than smaller ones. An intuitive explanation is that large farmers have more proximity to knowledge channels like formal and informal extension services^30^. The labour share is increasing from the lowest to the highest decile. However, irrigation shows a reverse pattern. It indicates that the large farming units engage in intensive farming. A similar pattern prevails for all inputs for the unit value (output per land) across deciles (Fig. S2).

Among the inputs, the cost of fertilizer and labour are the principal ones, varying across deciles. The area of the decile depicts the share of fertilizer to the total value of inputs. We divide each decile in Fig. (S3) by the first decile. The ratio for fertilizer cost tends to decline over the first to tenth decile range. It implies that marginal farmers rely more on chemical fertilizer in production, which is the reverse for large farmers. A plausible explanation is that the knowledge of soil health through channels like formal and informal extension may not reach marginal farmers^30^. The Government of India's recently launched soil health card scheme aimed to cover all sections of the farming category. While the awareness for the scheme is high, indicating the benefits for high fertilizer-consuming crops such as paddy or cotton, careful planning is needed to obtain widespread benefits to agriculture and the environment in the country^31^. These include specially designed pilot projects, use of technology, reduction in the subsidy of nitrogenous fertilizers, doorstep delivery of micro-nutrients, and prioritized funding for the development of supply chain infrastructure^31^.

Regarding labour expenditure, deciles show a consistent increase except for a slight dip in the last decile (Fig. 2a). Higher deciles are likely to provide more scope for scaling up the operations that require more labour. On the other hand, for the lower deciles, hired labour perhaps is unaffordable. Hence, they resort to their own account work (self-labour) for farming^32,33^. It is crucial to assess the outcomes if we divide these inputs by land (Fig. 2c). For these indices, however, the pattern remains the same (Fig. 2b, c). It means more land productivity and lesser use of chemical fertilizers, while the amount of labour tends to increase.

Further, we define the aggregate usage of green inputs (biofertilizers, manure and biopesticides) to the total value of inputs as the sustainability component (SC). We compute the deciles of either the value of the output or its unit value (Fig. 2b, c). Across deciles, the confidence interval (at 95 per cent) of the mean is of homogeneous width, and its statistically significant, explaining a consistent and systematic variation across deciles. The ratio consistently rises for the first set of deciles, and the same behaviour is also valid for the second set. It is an important pattern that unravels the link between affordability and sustainability. A relevant issue is why the green ratio is lowest for the lower strata, even after standardizing it for the land size (Fig. 2c). Plausibly, switching over to green inputs relies on affordability and awareness. Therefore, a natural question is whether the agricultural extension service caters to marginal farmers. Further, a significant policy issue is how to provide green inputs to the lower strata at affordable prices.

### Affordability of inputs across farming households

**Figure 3:**
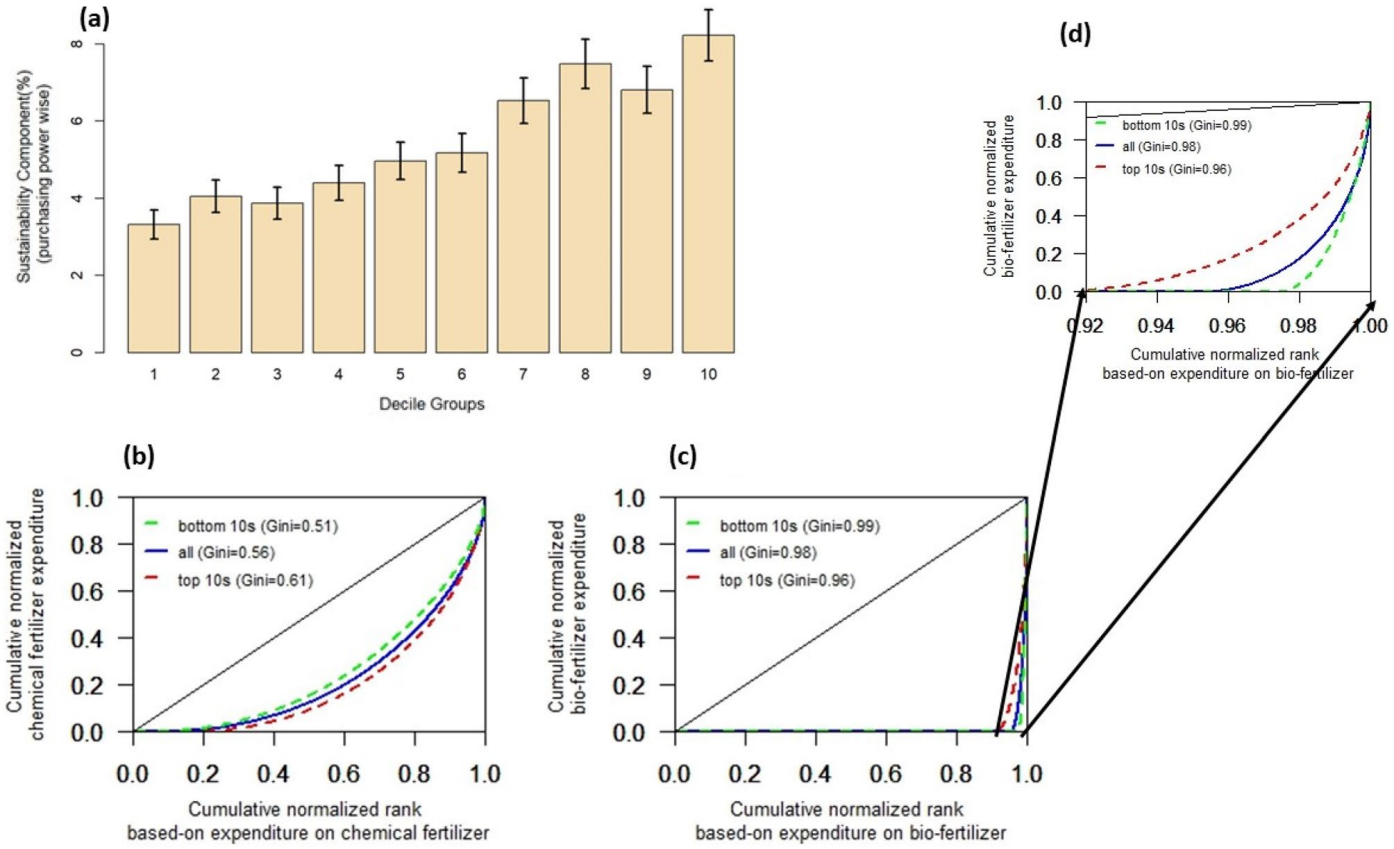
Distribution of sustainability component and measures of expenditure inequality on chemical and bio-inputs. Panel (**a**) shows the sustainability component arranged in deciles based on the MPCE (Monthly Per Capita Expenditure) values. The bar in panel (**a**) shows the average value, and the error bar shows the 95% confidence interval. Panel (**b**) shows the Lorenz curve for the expenditure on chemical fertilizer for the households with the bottom ten percentile (green dashed line), all households (solid blue line), and top 10 percentile (red dashed line) according to MPCE values. The legends in panel (**c**) and (**d**) are the same as in panel (**b**), except it shows the households’ expenditure on bio-fertilizers. Panel (**d**) is the zoomed portion of the panel (**c**) for better visualization of the legends. In panels (**b**), (**c**), and (**d**), the solid black straight line shows the one-to-one relationship of the cumulative rank and the cumulative values, which indicates the line of equality as per the description of the Lorenz curve. The Gini values in panels (**b**), (**c**), and (**d**) indicate the measure of inequality. The higher is Gini, the higher the inequality of distribution of a variable.

Regarding affordability, it is crucial to know if the SC varies across the economic strata, measured by the deciles of per capita monthly consumption expenditure (MPCE). For every decile, we compute the average of SC at a 95% confidence interval (Fig. 3a). There is a direct relationship between the economic strata of the farming unit and the SC adopted for farming, despite small dips at the third and ninth decile. The SC for the eighth decile is twice the first. It implies that adopting sustainability in farming is sensitive to the affordability of the farmers. Small and marginal farmers may find switching to green inputs difficult unless it is appropriately priced and supplemented by knowledge inputs. Considering that small and marginal farmers, in aggregate, substantially contribute to food production, the adoption of SC by them requires a comprehensive policy framework that considers affordability. On the other hand, an umbrella policy for SC that does not explicitly account for affordability may impact food security in the long run.

Further, to understand the distribution of expenditure on chemical and biological fertilizers in Indian farms, we used the Gini inequality index and the Lorenz curve^34^. Gini and Lorenz get widely adopted in literature to examine the income and wealth distribution in society. The Gini index takes values between 0 and 1. The closer the index is to 0, the more equal the distribution is and vice-versa^34^. The Lorenz curve shows the graphical distribution of income by the proportion of the society. We use Gini and Lorenz curve for the distribution of expenditure used on fertilizer (Fig. 3b) and io-fertilizer (Fig. 3c, d) per unit of land.

Interestingly, the expenditure distribution on chemical and bio-fertilizer is significantly differ (Figs. 3b–d). The results indicate that all MPCE-class farmers in India use chemical fertilizers. Although the inequality in expenditure in chemical fertilizer is high, the distribution of expense in chemical fertilizer is more homogenous among the lower MPCE class (poorest class of the farmers) compared to the wealthiest class of the farmers (Fig. 3b). These results confirm the findings of the village-level surveys, which indicate that the application of chemical fertilizer among poor farmers is quite prominent^35^. Field studies indicate that small farmers use high doses of fertilizer for cultivation, which often generates negligible returns subjected to climatic and market conditions^36^. Our results point out that a reduction in government subsidy on chemical fertilizer may have a detrimental economic impact on small farmers since their share in the use of chemical fertilizer is substantial and more homogenously distributed across households (Fig. 3b).

The distribution of biofertilizer looks sensitive to the tail of the distribution (Fig. 3c, d). The distribution is markedly skewed. It indicates that only the extremely rich farmers can afford and apply biofertilizers. Figure 3 (panels c and d) indicates that less than 5% of the farming population contributes to the 95% usage of bio-fertilizer in India. To ensure the long-term sustainability of Indian agriculture, bio-fertilizer distribution (Fig. 3c,d) needs to attain more equality across all sections of the farming population.

## Discussion

**Figure 4:**
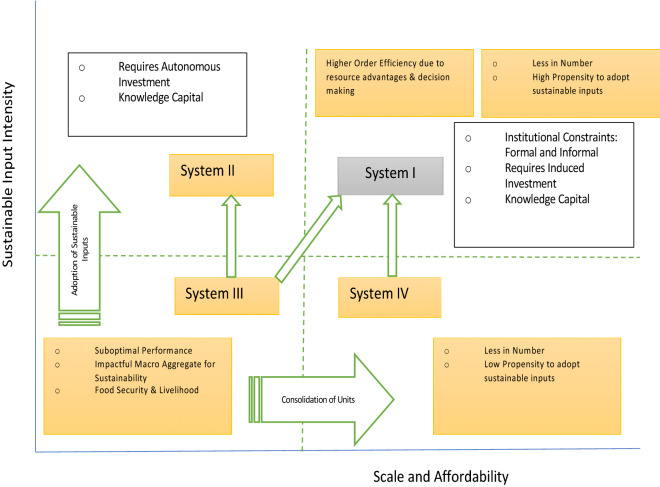
Systems of interaction between sustainable inputs in agriculture and scale of farming. The system refers to the way of organizing sustainable inputs given the scale of the land. System III is the baseline which characterizes the prevalent scenario, i.e., low use of sustainable inputs and fragmented lands, while the system I is the desired state depicting the upgrading. While system II is a more realistic outcome for countries like India owing to institutional constraints, system IV represents large-scale farming with low SC. Arrows represent the transition from one system to another. The shaded boxes depict the state of agriculture in each system, while the plain boxes convey the prerequisites for transformation.

It is crucial to evaluate the Indian farming system from two dimensions: scale and sustainable inputs (Fig. 4). The system consists of units that vary in the scale of operations. It ranges from highly marginal land holding to larger ones^37,38^. And the scale also corresponds to the order of the economic strata. The second dimension is the usage of sustainable inputs^39^. It also varies from low to high intensity. Juxtaposing these two generates ideas about the linkage between farming performance and the use of sustainable inputs. We slice the space into four quadrants called systems. The system I is a situation of medium to large-scale farming units and medium to high usage of sustainable inputs. What characterizes system II are small to medium-scale of farming and medium to high intensity of sustainable inputs. We get system III by combining small to medium scale and low to medium sustainable input usage. Finally, system IV consists of medium to large-scale operations and low to medium-intensity of sustainable inputs.

Each system has its specific features. System I is numerically small in Indian farm sector. What is highly probable in the current scenario is the positive link between scale and sustainable input usage. Induced investment and knowledge capital contribute to this^40^. Investment in technology is likely to generate better returns, considering the historically lower capital formation in Indian agriculture^41,42^. And large farming units may resort to it, contributing to efficiency gains^43^. It is crucial that knowledge capital, especially extension services, may go along with the propensity to adopt sustainable practices^30^. A major variant of this behaviour is the adoption of bio inputs over chemical inputs^44^. System II is less likely to exist and is futuristic. System III is the more common and numerically most significant category in India^45,46^. This system consists of the sub-optimal performance of production units^47^. Although a micro-unit in the system is of lower economic significance, the system as an aggregate is too crucial for the supply chain of food grain and food security. Moreover, it is the principal source of employment in the country. System IV is a less likely scenario.

There are three transition scenarios. First is the change from system III to II. Alternately, the second trajectory is from system III to I. The third transition is from IV to I. Although the second transition looks like a logical option, institutional constraints impede the journey, particularly in India^48^. Landholdings in India is not just a property right in the market^49,50^, it is also embedded with diverse social contexts such as joint family. Any initiative to unitize the land and consolidate is likely to meet with resistance from social forces and formal and informal institutions^51,52^. Therefore, the first trajectory is the feasible one. It implies that the transition involves the same scale of farming with more sustainable inputs, like prioritising organic inputs in rainfed and hilly regions that tend to use fewer chemical inputs.

Given that most farming units are marginal, they will require more capacity for investing in the transition to sustainable inputs. It calls for investments autonomous of returns by agencies like the state. A blanket policy on adopting sustainable inputs that is neutral on the scale may not work for the transition^53–55^. It is crucial to note that the above-discussed dynamics is a scenario of upgrading but not upscaling. However, the transition from system III to I, is a case of upscaling through institutional arrangements like a contract or corporate farming. Its political economy is a contentious issue in contemporary India^56^. An interesting scenario is a transition from IV to I. It involves motivating medium to large units to use sustainable inputs, primarily through induced investment, with a clear expectation of future return.

More succinctly, these transition paths depict heterogeneous contexts that call for appropriate policies to promote the use of sustainable inputs in farming. The transition to sustainability is sensitive to the social structure of knowledge creation and diffusion in farming systems. In India, the formal channel of knowledge consisting of government agencies and universities is less efficacious in impacting the decision to reduce chemical fertilizer. On the other hand, the private channel, including progressive farmers, commercial agents, and non-governmental organizations, are impactful in decisions to reduce the use of chemical fertilizer^30^. Without understanding the heterogeneity of transition, a policy favouring wide adoption as a standard template may trigger undesirable outcomes, especially food security^55^. If the policy confides in induced investment by the units and the scaling up as a route to promote sustainable input use, it is unlikely to motivate the marginal units. From a micro perspective, it is merely a decision problem by the producer. However, its macro dimension is rather complex since the aggregate of these units translates to significant stakes in the public distribution system and livelihoods.

## Data and methods

### Data

We use the microdata from the National Sample Survey 77th round (NSS 77th round) survey on the theme of “land and livestock holding of households and situation assessment of agricultural households”. National Statistical Office conducts the survey, Government of India. The data was collected during 2018–2019, which captured the information for two cropping seasons. There were separate visits for both seasons. While the first round captured the data for July to December 2018 (monsoon season), the second round was from January to June 2019 (post-monsoon season). Since Indian agriculture is predominantly rainfed, we examine only the data from the monsoon season. The farming household is the unit of analysis. Either the head of the household or a key informant (a representative of the household familiar with farming details) is the respondent. Samples were drawn from 5940 first-stage units (hamlet groups). Our sample comprises 58,035 households across India (except for the union territory Andaman and Nicobar Islands).

### Variables

Fundamentally the analysis examines the monetary value of output and input used in agriculture. Further, these measures are also divided by the area of land operated for farming (in ha). The value of output refers to the monetary value of the output produced. The value of the input is the sum of the monetary value of diverse components. These components include chemical fertilizers, manure, biofertilizers, chemical pesticides, biopesticides, labour, irrigation, crop insurance, and other inputs. Chemical fertilizers are either inorganic materials or synthetic ones. It supplies nutrients to the growth of plants. For example, ammonium sulphate, nitrate, phosphate, and urea are chemical fertilizers. Biofertilizers have living microorganisms that contribute to the growth of the crop, for example, *Rhizobium*, *Bacillus sp.,* and *Mycorrhiza*. Manure is a natural substance that emerges from the waste of plants and animals, for example, cow dung. Pesticide refers to chemical plant protection material like Copper Sulphate or Lime-Sulphur. Biopesticides are non-chemical plant protection materials like *Azadirachta indica* (Neem oil), *Brassica napus* (Rapeseed oil) and *Mentha piperita* (Mint oil).

Labour costs are the value of payments to the hired labour irrespective of the nature of the contract (be it regular or casual employment). Irrigation, crop insurance, and other inputs are valued per actual expenses incurred. Apart from the value of inputs and output, we analyse the monthly consumption expenditure of the household. It is divided by the size of the households to arrive at monthly per-capita consumption expenditure. This variable captures regular spending on durables and non-durables incurred by the household. And it is a proxy for the economic well-being of the household.

### Methods

We deploy a descriptive approach to dissect the variables of interest. It includes the comparison across quartiles and deciles. In quartiles, three values split the sorted data into four parts, each with an equal number of observations. The lowest quartile is the bottommost strata (lowest 25% of the data), while the highest quartile is the topmost strata (highest 25%). To visualize the quartile, the box plot is used. If an observation lies outside the box, it is called an outlier, situating in either extreme. Within the box, the median is the crucial indicator of central tendency used for comparison across quartiles. It is crucial to analyze the deciles for incisive data slicing. Decile implies that data is split into ten equal-sized bins with nine cut points. Its utility lies in a more microscopic assessment of tails. We compute the median for every decile and divide ith decile ${D}_{i}\left(\overline{x }\right)$ by the first decile ${D}_{1}\left(\overline{x }\right)$, called the multiplier (${M}_{i}$). Equations (1)–(3) describe computing.

1 $${D}_{i}=i\times \frac{\left(n+1\right)}{10}$$

2 $${D}_{i}\left(\overline{x }\right)=Median\, for\, {D}_{i}$$

3 $${M}_{i}=\frac{{D}_{i}\left(\overline{x }\right)}{{D}_{1}\left(\overline{x }\right)}$$

where $i$ is the order of the decile (1 to 10); $n$ is the number of sorted and ungrouped observations, and $D$ is a particular decile. *D* ($\overline{x }$) is the average of the decile. The purpose of the multiplier is to convey the volume of growth or contraction in the average across the distribution, taking the first decile as the reference point. For example, suppose it is a case of growth; the multiplier informs about the particular decile at which the first decile doubles, trebles, or quadruples. It is valid for contraction as well. We also compute a sustainability component (SC) indicator. SC is the expenditure on biofertilizer, manure and biopesticide as a proportion of the total input expenditure. The higher the decile average value {${D}_{i}\left(\overline{x }\right)\}$ of SC, the greater the orientation towards environmental sustainability and vice versa. Here, we use the average instead of the median because the mode is closer to zero. Another crucial reason for using the average is to examine if the variation is consistent across deciles. It can be gauged by computing the confidence interval at a suitable level.

## Supplementary Information

Supplementary Information.

## Data Availability

The datasets used and/or analysed during the current study available from the corresponding author on reasonable request.

## References

[1] Sachs, J., Lafortune, G., Kroll, C., Fuller, G. & Woelm, F. *From Crisis to Sustainable Development: the SDGs as Road Map to 2030 and Beyond*. Sustainable development report (Cambridge University Press, 2022). 10.1017/9781009210058

[2] Zhang, X. & Davidson, E. *Sustainable Nitrogen Management Index (SNMI): Methodology (University of Maryland Centre for Environmental Science, 2016)*, Available from: https://www.umces.edu/sites/default/files/profiles/files/Ranking%20Method_submit_to_SDSN_SNMI_20160705_0.pdf.

[3] Economic Survey. Economic Survey 2021–22 (New Delhi: Government of India, 2022)

[4] Chand, R. & Singh, J. Workforce changes and employment, *NITI Aayog Discussion Paper*, (NITI Aayog, Government of India, 2022), Available from: https://www.niti.gov.in/sites/default/files/2022-04/Discussion_Paper_on_Workforce_05042022.pdf

[5] Tripathy, S. Farmers, climate change and people centric disaster management in India in (eds Malhotra, V. K., Fernando, R. L. S. & Haran, N. P.). *Disaster Management for 2030 Agenda of the SDG. Disaster Research and Management Series on the Global South* 181–195 (Palgrave Macmillan, Singapore, 2020) 10.1007/978-981-15-4324-1_13

[6] Foster AD, Rosenzweig MR (2010). Microeconomics of technology adoption. Annu. Rev. Econ..

[7] Pahalvi, H. N., Rafiya, L., Rashid, S., Nisar, B. & Kamili, A. N. Chemical fertilizers and their impact on soil health. In *Microbiota and Biofertilizers*. (eds. Dar, G. H., Bhat, R. A., Mehmood, M. A. & Hakeem, K. R.), **2** (Springer, Cham, 2021). 10.1007/978-3-030-61010-4_1

[8] Tahat M, Alananbeh MK, Othman A (2020). Soil health and sustainable agriculture. Sustainability.

[9] Laishram J, Saxena KG, Maikhuri RK, Rao KS (2012). Soil quality and soil health: A review. Int. J. Ecol. Environ. Sci..

[10] Bai YC (2020). Soil chemical and microbiological properties are changed by long-term chemical fertilizers that limit ecosystem functioning. Microorganisms.

[11] Li DP, Wu ZJ (2008). Impact of chemical fertilizers application on soil ecological environment. J. Appl. Ecol..

[12] Ferroni M, Zhou Y (2012). Achievements and challenges in agricultural extension in India. Glob. J. Emerg. Mark. Econ..

[13] Vandendriessche H, Bries J, Geypens M (1996). Experience with fertilizer expert systems for balanced fertilizer recommendations. Commun. Soil Sci. Plant Anal..

[14] Ramaswami B (1992). Production risk and optimal input decisions. Am. J. Agric. Econ..

[15] Cassman KG, Grassini P (2020). A global perspective on sustainable intensification research. Nat. Sustain..

[16] Cui Z (2018). Pursuing sustainable productivity with millions of smallholder farmers. Nature.

[17] Atieno M (2020). Assessment of biofertilizer use for sustainable agriculture in the Great Mekong Region. J. Environ. Manag..

[18] Brahmaprakash GP, Sahu PK (2012). Biofertilizers for sustainability. J. Indian Inst. Sci..

[19] Joshi NS, Parmar VS, Prajapati PJ, Kachhadiya NM, Hadiya NJ (2019). Constraints faced by farmers in adoption of bio fertilizer. J. Pharmacogn. Phytochem..

[20] Bagyaraj, D. J. Quality control and constraints in biofertilizer production technology. In *Biofertilizers Technology* (eds. Kannaiyan, S., Kumar, K. & Govindarajan, K.) 401–408 (Scientific Publishing, India, 2004)

[21] Ghosh TK, Singh RP, Duhan JS, Yadav DS (2001). A review on quality control of biofertilizer in India. Fertil. Mark. News.

[22] DAFM. in *Department of Agriculture and Farmer Welfare Annual Report 2021–22, Ministry of Agriculture and Farmers Welfare* (Government of India, New Delhi, 2022)

[23] Reddy, A. A. *Impact study of Paramparagath Krishi Vikas Yojana* (*Organic Agriculture)* (MANAGE), Rajendranagar, Hyderabad–500030. Telangana State, I. 81 pp. Preprint Available from: https://www.manage.gov.in/publications/reports/pkvy.pdf

[24] Seufert V, Ramankutty N, Foley JA (2012). Comparing the yields of organic and conventional agriculture. Nature.

[25] Barman M, Paul S, Choudhury AG, Roy P, Sen J (2017). Biofertilizer as prospective input for sustainable agriculture in India. Int. J. Curr. Microbiol. Appl. Sci..

[26] Khurana, A. & Kumar, V. State of biofertilizers and organic fertilizers in India (New Delhi: Centre for Science and Environment, 2022), Available from: https://www.cseindia.org/state-of-biofertilizers-and-organic-fertilizers-in-india-11235

[27] NSSO. in *Situation Assessment of Agricultural Households and Land and Holdings of Households in Rural India, 2019* (Ministry of Statistics and Programme Implementation, Government of India, New Delhi, 2021)

[28] Jayaraman T, Murari KK (2014). Climate change and agriculture: Current and future trends, and implications for India. Rev. Agrar. Stud..

[29] Swaminathan, M. S. *et al.* Serving farmers and saving farming 2006: Year of agricultural renewable, fifth and final report (National Commission of Farmers, Government of India, 2006), Available from: https://agricoop.nic.in/sites/default/files/NCF5%20Vol.-1%20%281%29.pdf

[30] Paul B, Patnaik U, Sasidharan S, Murari KK, Bahinipati CS (2022). Fertilizer use, value, and knowledge capital: A case of Indian farming. Sustainability.

[31] Reddy AA (2019). The soil health card Scheme in India: Lessons learned and challenges for replication in other developing countries. J. Nat. Resour. Policy Res..

[32] Patnaik U, Das PK (2017). Do development interventions confer adaptive capacity? Insights From rural India. World Dev..

[33] Sen A (1981). Market failure and control of labour power: Towards an explanation of structure and change in Indian agriculture. Part 1. Camb. J. Econ..

[34] Sitthiyot T, Holasut K (2021). A simple method for estimating the Lorenz curve. Humanit. Soc. Sci. Commun..

[35] Murari KK, Jayaraman T, Swaminathan M (2018). Climate change and agricultural suicides in India. Proc. Natl Acad. Sci. USA.

[36] Murari, K. K. Fertilizer use in West Bengal: a case study of three villages in Socio-economic Survey. In: *Three Villages of West Bengal: A Study of Agrarian Relationship* (eds Bakshi, A. & Modak, T.S.), 171–195 (Tulika Books, New Delhi, 2022)

[37] Kumar S, Rao DUM, Thombare P, Kale P (2020). Small and marginal farmers of Indian agriculture: Prospects and extension strategies. Indian Res. J. Ext. Educ..

[38] Chand R, Prasanna PL, Singh A (2011). Farm size and productivity: Understanding the strengths of smallholders and improving their livelihoods. Econ. Pol. Wkly..

[39] Patil S, Reidsma P, Shah P, Purushothaman S, Wolf J (2014). Comparing conventional and organic agriculture in Karnataka, India: Where and when can organic farming be sustainable?. Land Use Policy.

[40] Bharucha ZP, Mitjans SB, Pretty J (2020). Towards redesign at scale through zero budget natural farming in Andhra Pradesh. India. Int. J. Agric. Sustain..

[41] Dwivedi S, Sharma P, Bhat A (2011). An analytical study of capital formation in India: With special reference to Indian agriculture. Econ. Aff..

[42] Chand R, Kumar P (2004). Determinants of capital formation and agriculture growth: Some new explorations. Econ. Pol. Wkly..

[43] Mishra SN (1996). Capital formation and accumulation in Indian agriculture since independence. Indian J. Agric. Econ..

[44] Reddy, G. C. *et al.* Biofertilizers toward sustainable agricultural development. In *Plant Microbe Symbiosis *(eds. Varma, A., Tripathi, S. & Prasad, R.), (Springer, Cham, 2020). 10.1007/978-3-030-36248-5_7

[45] Dev, M. S. (2014). Small farmers in India: Challenges and opportunities. Available from: http://www.igidr.ac.in/pdf/publication/WP-2012-014.pdf

[46] Bardhan PK (1973). Size, productivity, and returns to scale: An analysis of farm-level data in Indian agriculture. J. Pol. Econ..

[47] Cornia GA (1985). Farm size, land yields and the agricultural production function: An analysis for fifteen developing countries. World Dev..

[48] Kalirajan KP, Shand RT (1997). Sources of output growth in Indian agriculture. Indian J. Agric. Econ..

[49] Rao N (2006). Land rights, gender equality and household food security: Exploring the conceptual links in the case of India. Food Policy.

[50] Patnaik U (1976). Class differentiation within the peasantry: An approach to analysis of Indian agriculture. Econ. Pol. Wkly..

[51] Albertus M (2020). Land reform and civil conflict: Theory and evidence from Peru. Am. J. Pol. Sci..

[52] Alston LJ, Libecap GD, Mueller B (2000). Land reform policies, the sources of violent conflict, and implications for deforestation in the Brazilian Amazon. J. Environ. Econ. Manag..

[53] Chand, R. Indian agriculture towards 2030-need for a transformative vision in (eds. Chand, R., Joshi, P. & Khadka, S.) *Indian Agriculture Towards 2030 India* 1–8 (Springer, Singapore, 2022). 10.1007/978-981-19-0763-0_1

[54] Gulati, A. & Juneja, R. Transforming Indian agriculture. In *Indian Agriculture Towards 2030 *(eds. Chand, R., Joshi, P. & Khadka, S.), 9–37 (Springer, Singapore, 2022). 10.1007/978-981-19-0763-0_2

[55] Reddy AA (2022). Economic impact of organic agriculture: evidence from a Pan-India survey. Sustainability.

[56] Singh, S. Contract farming for agricultural development: Review of theory and practice with special reference to India. In *Reforming Indian Agriculture: Towards Employment Generation and Poverty. Reduction essays in honour of GK Chadha* (ed. Bhaumik, S.K.)*.* 191–230 (SAGE, New Delhi, 2008).

