# Supplementary material for: Sustainability transition for Indian agriculture
Source: Sci Rep. 2023 May 5;13:7290. doi: 10.1038/s41598-023-34092-0 (PMC10162982; doi:10.1038/s41598-023-34092-0)
Supplement: Supplementary file 1 — Supplementary Information. [file 41598_2023_34092_MOESM1_ESM.docx]

**Supplementary Information**

**Sustainability Transition for Indian Agriculture**

**S1: The burden of inputs on farming households**

In Fig. S1, we split the farmers into four quartiles based on the value of the output. Box-whisker plots visualize each quartile for output and input values. In the first quartile (lowest), outliers dominate input distribution, showing discernibly higher dispersion (Fig. S1a). However, the distribution of output is compact without any outliers. The median values for both distributions are not visibly distinct. The striking behaviour emerging from the plot is marked skewness in input to produce output. In essence, varying input levels culminate in a narrow output range, leaving little scope for fortunes. It combines marginal farmers' precarious and subsistent living and a deterministic outcome.

Further, it defies the conventional notion of returns to scale. Another crucial aspect is the negligible margin generated in the process. The value of output is hardly distinct from that of input. It implies that the marginal farmer is unlikely to have an operating surplus that feeds into the future cycle. However, in the second quartile, we observe a small margin; the output value is slightly higher than the input cost (Fig. S1b). Although the input value shows a skewed distribution with some outliers, it is more compact than the lowest quartile. Here, the output value has no outlier, and the mean is visibly higher than the inputs. The pattern reveals that uptake from the marginal level to the next level reduces input dispersion while translating to a higher margin. Perhaps, the behaviour of this sort is well encrypted in neo-classical models that see the monotonic correspondence between the scale of the process and the performance. However, our investigation is towards the logic of relating performance and sustainability.


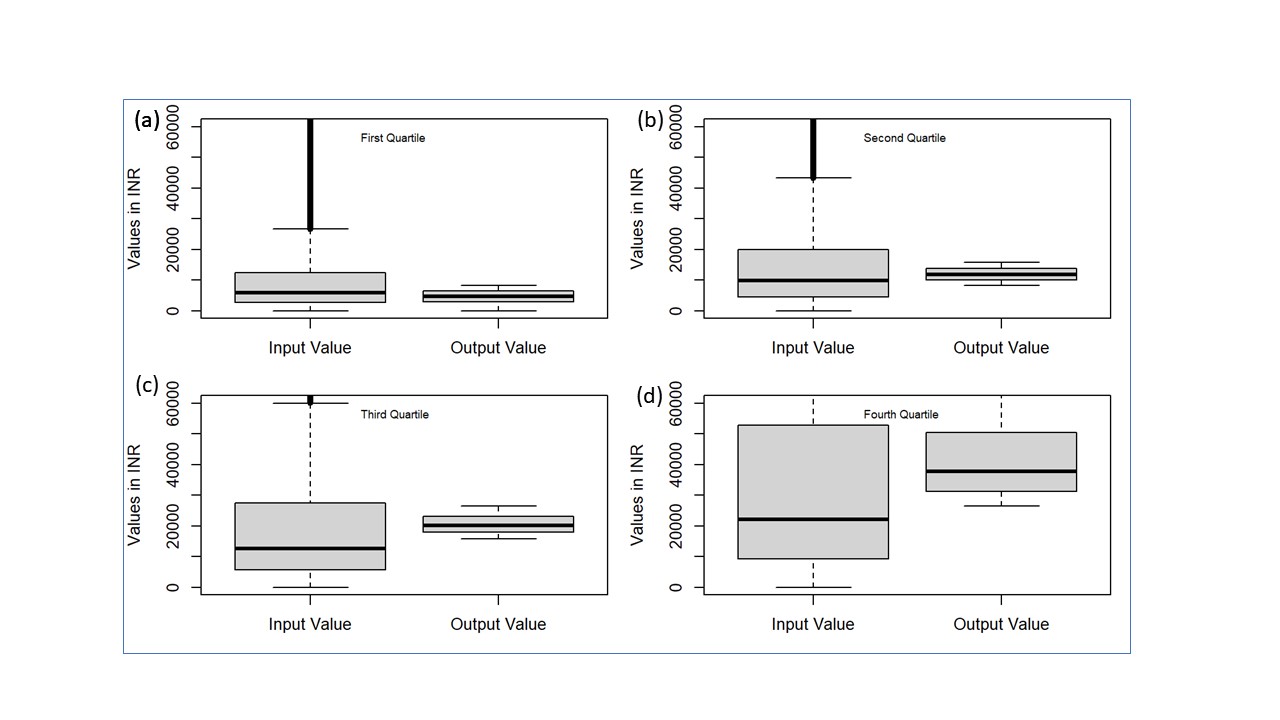


Fig. S1: Box and whiskers plot for inputs and GVO values for land cultivated across 58,035 farming households in NSS 77^th^ round microdata. The vertical axis in panels (a), (b), (c), and (d) are values (in INR) of total household level input and GVO values.

Interestingly, the third quartile is profoundly distinct (Fig. S1c). First, the distribution of input has few outliers. Second, the margin of output value over input is substantially higher, and it is almost a tip-off from the trap of low margin and higher input dispersion. The pattern in the third quartile resembles the conventional story of a monotonic relation between input and output. Until now, the story was about either the pair of the skewed input distribution with no margins or a relatively homogeneous input distribution with some margin. With the highest quartile, however, the scenario changes (Fig S1d). It involves a combination of an input distribution without any outliers and a higher margin between output and input. However, the input distribution’s upper limit is somewhat opaque. The opacity in input distribution seems to have resulted from more possibilities in farming activities that exist on a larger scale.

**S2: Different input use across household classes**

**
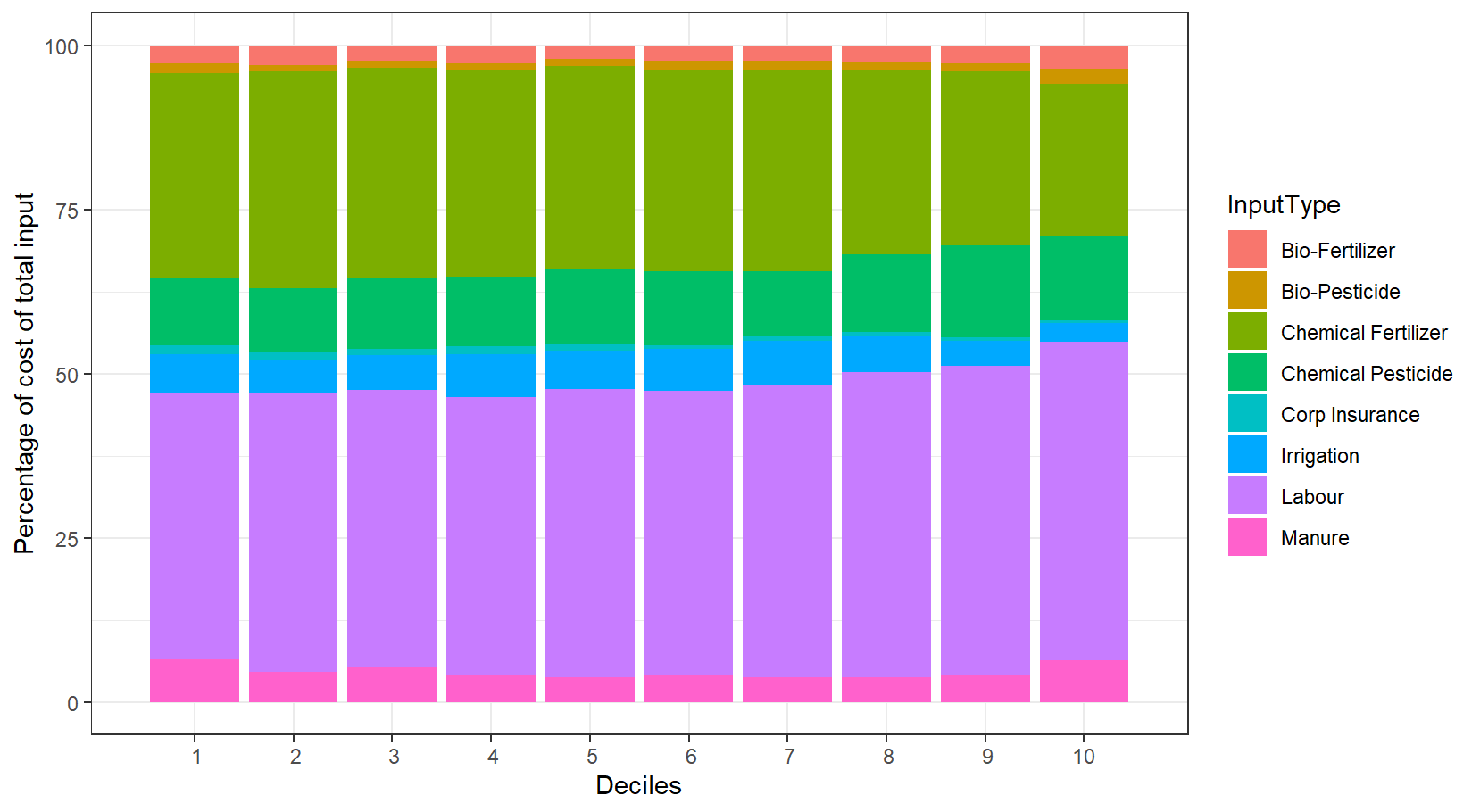
**

**Fig. S2:** Input-use in Indian farming. The figure shows the relative cost of different components of input costs. Note that the figure shows only the cost of inputs, labour, crop insurance, and irrigation. The figure does not consider other inputs such as seeds, machines, land rent, and miscellaneous costs. The value indicates the median values of the farming households in NSS 77th round.


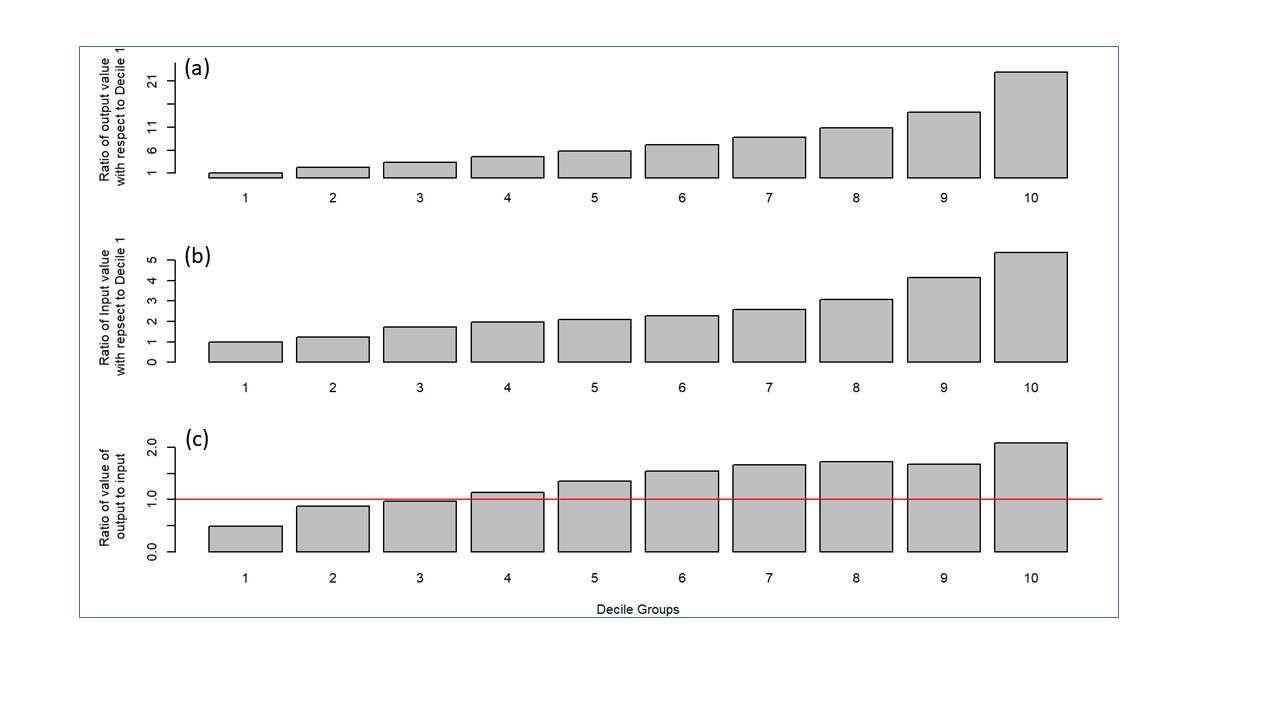


**Fig. S3:** Comparison of different deciles of GVO classes. Panel (a) shows the ratio of median values of GVO of each decile to decile 1. Decile groups are classed according to the GVO values of the households. Panel (b) shows the ratio of median values of the input cost of decile classed to decile 1. Panel (c) shows the median values of ratios of GVO to input cost for each decile class.

It is clear from panel (c) that the ratio is very close to or lower than one till the first five deciles, indicating that the marginal input costs are higher for the marginal, small, and medium farmers showing negative returns from the agricultural activities. However, the signature of negative returns is not clear in the sixth to last decile classes, indicating that these classes of farmers are getting a fair number of returns from agricultural activities. Except for the higher decile, no farming class gets a return double their input cost. It is an interesting observation, particularly in light of the ongoing talk about doubling the farming income by 2023, which seems only plausible for the wealthy class of farmers.
